# HMMER web server: 2026 update

**DOI:** 10.1093/nar/gkag373

**Published:** 2026-04-27

**Authors:** Aleksandar Rajković, Martin Beracochea, Alexander B Rogers, Sean R Eddy, Nicholas P Carter, Robert D Finn

**Affiliations:** EMBL-EBI European Bioinformatics Institute, Wellcome Trust Genome Campus, Hinxton, Cambridge CB10 1SD, United Kingdom; EMBL-EBI European Bioinformatics Institute, Wellcome Trust Genome Campus, Hinxton, Cambridge CB10 1SD, United Kingdom; EMBL-EBI European Bioinformatics Institute, Wellcome Trust Genome Campus, Hinxton, Cambridge CB10 1SD, United Kingdom; Department of Molecular & Cellular Biology, Harvard University, Cambridge, MA 02138, United States; Department of Molecular & Cellular Biology, Harvard University, Cambridge, MA 02138, United States; EMBL-EBI European Bioinformatics Institute, Wellcome Trust Genome Campus, Hinxton, Cambridge CB10 1SD, United Kingdom

## Abstract

The HMMER web server, available at https://www.ebi.ac.uk/Tools/hmmer, provides online access to tools from the HMMER software suite (http://hmmer.org/) for protein analysis using profile hidden Markov models. Users can perform sequence similarity searches against a range of regularly updated protein sequence databases or annotate protein sequences with domains and families using profile HMM libraries from protein family databases. Since the 2018 update, the continued exponential growth of sequence databases has necessitated substantial infrastructural improvements to maintain search performance speed and service reliability. To achieve this, the web interface has been completely reengineered using modern web technologies (JavaScript and React), providing users with an enhanced experience, including session-based search history and streamlined results visualization. The web application programming interface has been rewritten to better support programmatic access with updated endpoints and JSON-based responses. The infrastructure has been redesigned to efficiently handle searches against much larger databases through horizontal scaling and asynchronous job processing. Target database offerings have been updated to reflect current usage patterns and data availability. The HMMER web server is free and open to all users, and there is no login requirement.

## Graphical Abstract

**Graphical Abstract:**
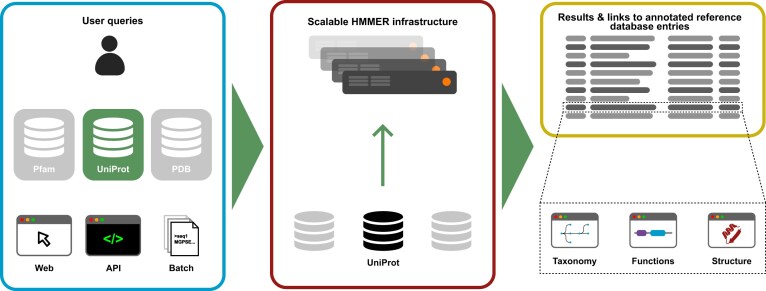

## Introduction

The HMMER web server provides a convenient interface to the HMMER software suite for biosequence analysis using profile hidden Markov models (HMMs) [1]. HMMER implements sensitive homology searching and sequence annotation methods based on probabilistic inference and has been widely applied to tasks ranging from protein family classification to metagenomic sequence annotation. The web server, first launched in 2011 and last described in 2018 [2], offers four principal search modes: phmmer (protein sequence versus protein sequence database), hmmsearch (profile HMM versus protein sequence database), hmmscan (protein sequence versus profile HMM database for functional annotation), and jackhmmer (an iterative search against a protein sequence database). The search modes phmmer, hmmsearch, and hmmscan were introduced and described in the original HMMER web server publication [3], while jackhmmer was subsequently described in the update paper [4]. Briefly, phmmer enables the searching of a protein sequence against a protein target database. Hmmsearch enables searching of a profile HMM (either submitted directly or calculated based on a protein multiple sequence alignment) against a protein target database. Jackhmmer enables iterative searching, which can be initiated with a protein sequence, a multiple sequence alignment, or profile HMM against a target protein database. Finally, hmmscan enables the searching of a protein sequence against a profile HMM library.

The landscape of computational biology has evolved considerably in recent years, with the development of sequence search tools like MMseqs2 [5] and deep learning-based methods such as NEAR [6], which offer additional capabilities for large-scale protein sequence analysis. Protein language models such as ESM [7] and ProstT5 [8] have similarly emerged as powerful new tools for protein function annotation. Nevertheless, profile HMM-based homology searching remains a foundational tool for sequence analysis. Profile HMM methods provide interpretable alignments, quantifiable statistical significance, and transparent evolutionary models that enable the explanation of the match between the query and the target, unlike many of the deep learning approaches. Profile HMMs excel at detecting remote homology and remain the method of choice for systematic protein family classification and domain annotation in resources such as Pfam [9], InterPro [10], and the UniProt Knowledgebase (UniProtKB) [11]. The HMMER web server currently receives approximately 500 000 searches per year from around 62 000 unique visitors per month, demonstrating sustained demand for profile HMM-based sequence analysis via the web interface.

While the core algorithms within the HMMER suite have remained stable since the introduction of HMMER3, the engineering challenge of maintaining a responsive web service has grown substantially. The early implementations relied on storing the reference sequence database, an amalgamation of all target databases, in memory, but the reference sequence databases have continued to grow substantially: UniProtKB, for example, has more than doubled in size since our 2018 update, now exceeding 250 million sequences, while metagenomic sequence databases have billions of protein sequences [12]. This growth places increasing demands on search infrastructure. This update therefore focuses primarily on the substantial architectural improvements designed to maintain fast search performance and high availability as database sizes continue to grow.

## Infrastructure and scaling

### The scaling challenge: growing reference databases

The primary technical challenge facing the HMMER web server in recent years has been the continued exponential growth of protein sequence databases, impacting computational performance as both search times and memory requirements scale linearly with the database size. Furthermore, as the number of significant matches typically grew proportionally with the database growth, additional limitations were found when processing the HMMER search results for display on the website. To address this, we have implemented a major improvement for scaling the in-memory database and prevented the saturation of web server connections caused by long search times via the introduction of an additional search queue.

### Horizontal scaling with *hmmpgmd_shard*

The original HMMER web server architecture relied on *hmmpgmd*, a long-running program deployed across multiple virtual machines, with one virtual machine acting as a master node coordinating searches and the rest serving as worker nodes. While this approach offered excellent performance, horizontal scaling became prohibitively expensive, as each node must load the complete database. Thus, the previous infrastructure, deployed in 2018, consisted of 16 dedicated virtual machines, each with 78 GB of RAM. However, the memory requirements for loading complete databases were approaching hardware limits, and the monolithic architecture offered limited flexibility for load balancing or fault tolerance. To address this limitation, *hmmpgmd_shard* has been developed, a variant designed to operate on database partitions called shards. It was made available to the public with the release of HMMER 3.3 (https://github.com/EddyRivasLab/hmmer/releases/tag/hmmer-3.3). In the case of *hmmpgmd_shard*, the target database is divided into *n* approximately equal-sized chunks, with each worker searching only its assigned partition. When a user submits a query, the search is dispatched to all *n* worker nodes in parallel. The results are collected and merged by the master node that performs the final *E*-value calculations. This sharding strategy renders the database scanning problem more parallelizable, while maintaining acceptable response times as database sizes grow. Furthermore, rather than having a single reference sequence database, the target databases have been partitioned into distinct sets, with a set of *hmmpgmd_shard* nodes dedicated to running each amalgamated set of target databases. For example, all UniProt-derived sequence databases are merged into a single set, while PDB represents another set. This modular design allows independent deployment and updates according to each database’s release cadence (e.g. PDB is updated every two weeks, while UniProt databases are updated approximately every two months), simplifies maintenance and versioning, and enables targeted resource allocation.

The current deployment spans approximately 20 nodes, each with 64 GB of RAM, and the use of Kubernetes enables elastic resource allocation that can scale dynamically in response to demand. The containerized architecture has enabled standardization of the deployment environment and provides built-in service discovery, load balancing, and automated restart of failed components.

### Asynchronous job queue

**Figure 1.**
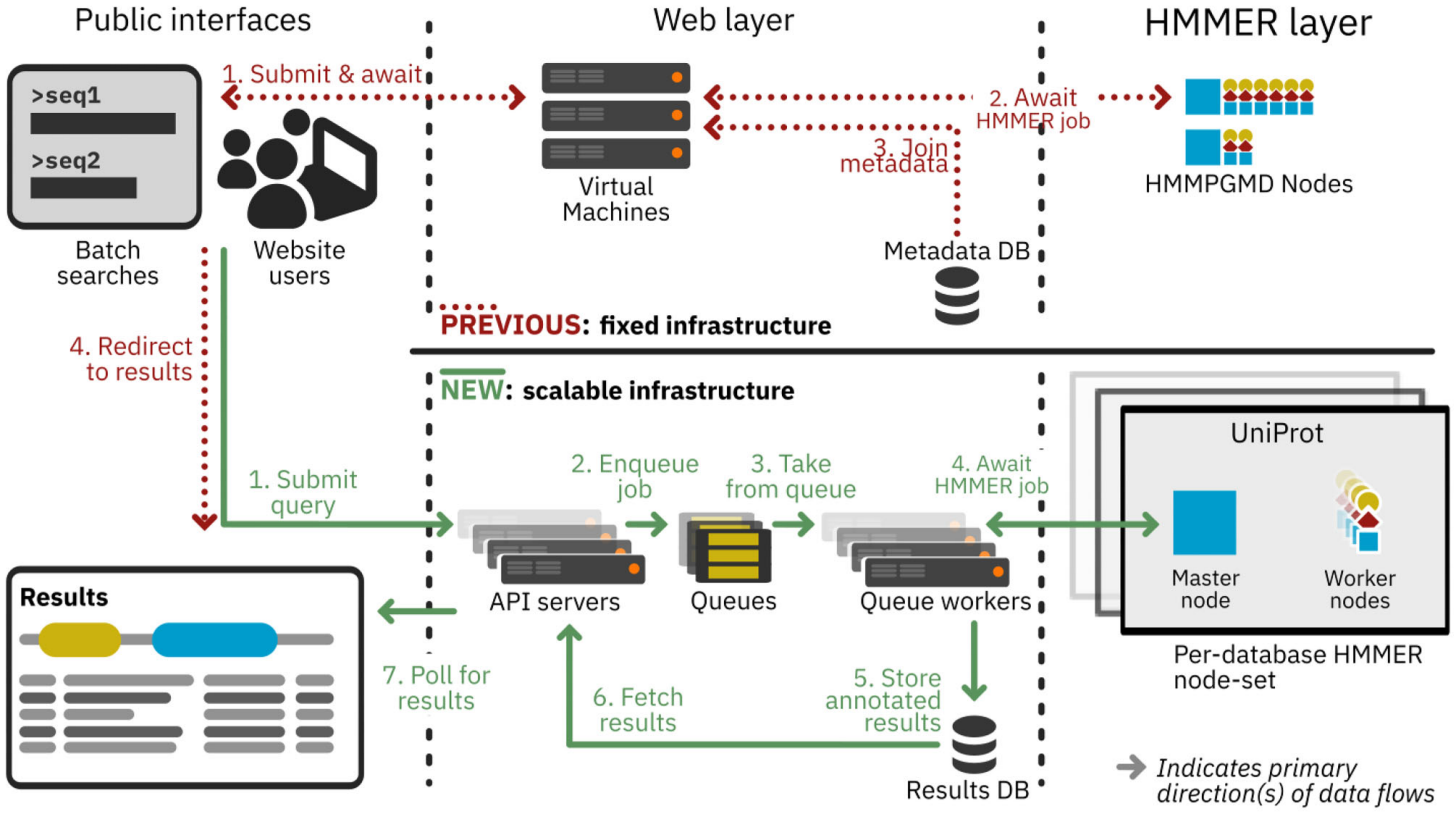
Architecture and data-flow diagram of the previous (top, red dashed arrows) and new (bottom, green solid arrows) infrastructures. Both single and batch queries continue to be supported, and the results display is a modernized equivalent of the previous interface. Major changes include introducing dynamic scalability throughout the web and HMMER compute layers, asynchronicity via work queues and a results database, and dedicated node-sets in the HMMER layer for different target databases. In the new infrastructure, the metadata of each target sequence is stored alongside the sequences to avoid joining to a separate database.

The interaction model between the web server and search infrastructure has been redesigned to use an asynchronous job queue. In the previous architecture (Fig. 1), the web server communicated directly with *hmmpgmd* daemons via TCP sockets on separate virtual machines. The web server would block while awaiting a response from the daemons, then immediately return results to the user. While this synchronous model offers simplicity and low latency, it presented challenges for robustness and resource management. More critically, this approach did not scale effectively beyond 40 concurrent users or when searches were taking longer than 5 min, meaning that any additional new search request would not yield results within an acceptable time window, causing the client to time out and the search to be “lost.” The new architecture decouples the web server from the search daemons via a message queue managed by Celery (https://github.com/celery/celery) (labeled “Queues” in Fig. 1). When a user submits a search, the web server queues the job and returns a job identifier to the user immediately. Celery workers retrieve jobs from the queue, communicate with the appropriate *hmmpgmd_shard* master node, write results to persistent storage, and notify the front end about the job completion. To mitigate the latency introduced by this asynchronous approach, the interface provides progress indicators and status updates, and users can optionally provide an email address to receive notifications when their search completes. Failed searches are automatically retried, and the web server remains responsive during high load. While searches against large databases may take tens of seconds or even minutes, users can navigate away and return later.

The batch search functionality allows users to submit multiple sequences both through the application programming interface (API) and the web interface via a file. This feature has also benefited from the new asynchronous infrastructure. Historically, such batch submissions caused heavy system loads as the searches were interleaved with interactive searches, often resulting in performance degradation with the previous architecture. The current design enables more effective load balancing and resource allocation, allowing batch jobs to be processed in a more controlled fashion without affecting the responsiveness of the web interface even when queue volumes are high. Additionally, the increased system visibility allows for dynamic adjustment of queue sizes and priorities. Our initial deployment allows the *queue workers* (Fig. 1) for interactive queries to be scaled up to a relative priority three times higher than batch queries; however, if we observe increasing latencies for interactive queries, a configuration change can be automatically rolled out to deprioritize batch queries. Within the daemons, queries are parallelized as described in the original HMMER web server publication [3].

These improvements are reflected in measurable gains in service reliability. Average time between failures has increased significantly, with the current infrastructure achieving uptime in excess of 99.5% over the last 3 months of 2025. The combination of horizontal scaling, automated restarts, and continuous monitoring has substantially reduced the frequency and duration of service disruptions.

## Web client and API

### Modernized web API

The API of the server has been comprehensively rewritten in Python, replacing the previous Perl-based implementation. The new API provides JSON-formatted responses for all operations, including job submission, status polling, and results retrieval. Batch submission is supported, allowing users to queue multiple searches in a single request. As before, the API design follows RESTful principles, with resource-oriented endpoints and standard HTTP methods.

While maintaining backward compatibility was a design goal, the endpoint structure has changed in this version to better align with modern web API conventions. The new implementation is substantially easier to extend with additional features, and the entire API is now documented via an OpenAPI schema (https://www.ebi.ac.uk/Tools/hmmer/api/v1/docs). This schema serves as both human-readable documentation and a machine-readable specification that facilitates the automated generation of client libraries in multiple programming languages, lowering the barrier for programmatic access to HMMER searches.

### Updated web interface

**Figure 2.**
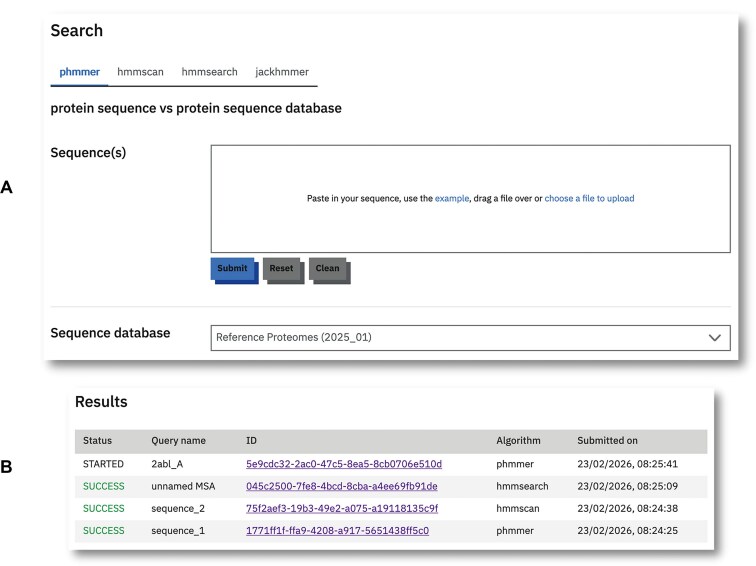
Screenshots of the updated web interface. (**A**) The updated input form with drag-and-drop capabilities. (**B**) The search history table showing previous search results and searches that are still in progress.

The web interface has been completely rebuilt using React and custom JavaScript components, sharing a layout, design, and styling framework consistent across many other EMBL-EBI hosted services [13]. Several usability improvements have been introduced in the new interface. Users can now submit searches via drag-and-drop (Fig. 2A) when uploading sequence files. The domain architecture visualizations have been redesigned to improve clarity while maintaining the information density users expect from HMMER results. A notable new feature is session-based search history (Fig. 2B), which allows users to review and retrieve results from previous searches within the same browser session. This is particularly useful for iterative analyses where users may wish to compare results across multiple queries or return to earlier searches without needing to bookmark individual result URLs. The search history is maintained client-side and does not require user authentication.

The web server codebase, comprising the frontend (https://github.com/EBI-Metagenomics/hmmer-app) and backend (https://github.com/EBI-Metagenomics/hmmer-api), is available as open source.

## Target databases

### Changes to database support since 2018

In this implementation of the HMMER web server, we have removed support for the reference databases Ensembl [14], MEROPS [15], ChEMBL [16], and Quest for Orthologs [17], as well as the profile HMM libraries CATH-Gene3D [18], TIGRFAMs [19], Superfamily [20], PIRSF [21], and TreeFam [22], all of which were available in the 2018 release. The decision to discontinue support for these databases and libraries was driven by two distinct considerations. Some databases, such as Quest for Orthologs, witnessed very low usage on the HMMER web server compared to other offerings (a median of 12 searches per month, with 90% of months seeing fewer than 64 searches), while others, such as TreeFam and MEROPS, are no longer actively maintained. By focusing on a smaller set of high-quality, well-maintained databases, we can dedicate more effort to ensuring their recency and optimizing search performance.

Currently, for searches targeted at sequence databases (phmmer, hmmsearch, and jackhmmer search algorithms), the server now provides access to UniProtKB, UniProtKB/Swiss-Prot (the manually curated section of UniProt Knowledgebase), UniProt Reference Proteomes (a set of representative proteomes) [11], and PDB (sequences from structures deposited in the Protein Data Bank) [23]. For domain annotation via hmmscan, the Pfam [9] database of curated protein families remains the primary resource.

Database processing and formatting is managed via automated Nextflow [24] pipelines, which handle the download, validation, and conversion of source databases into the *hmmpgmd*-compatible format required for HMMER searches. This workflow automation ensures reproducibility and simplifies the integration of updated database releases. The use of Nextflow allows us to standardize the processing steps across different database sources, manage complex dependencies, and facilitate maintenance as upstream database formats change. The pipeline is publicly available at https://github.com/EBI-Metagenomics/hmmer-db-pipeline, allowing users to run their own *hmmpgmd* instances against standard databases such as UniProt or adapt the workflow to work with locally curated sequence databases.

### Simplified metadata

**Figure 3.**
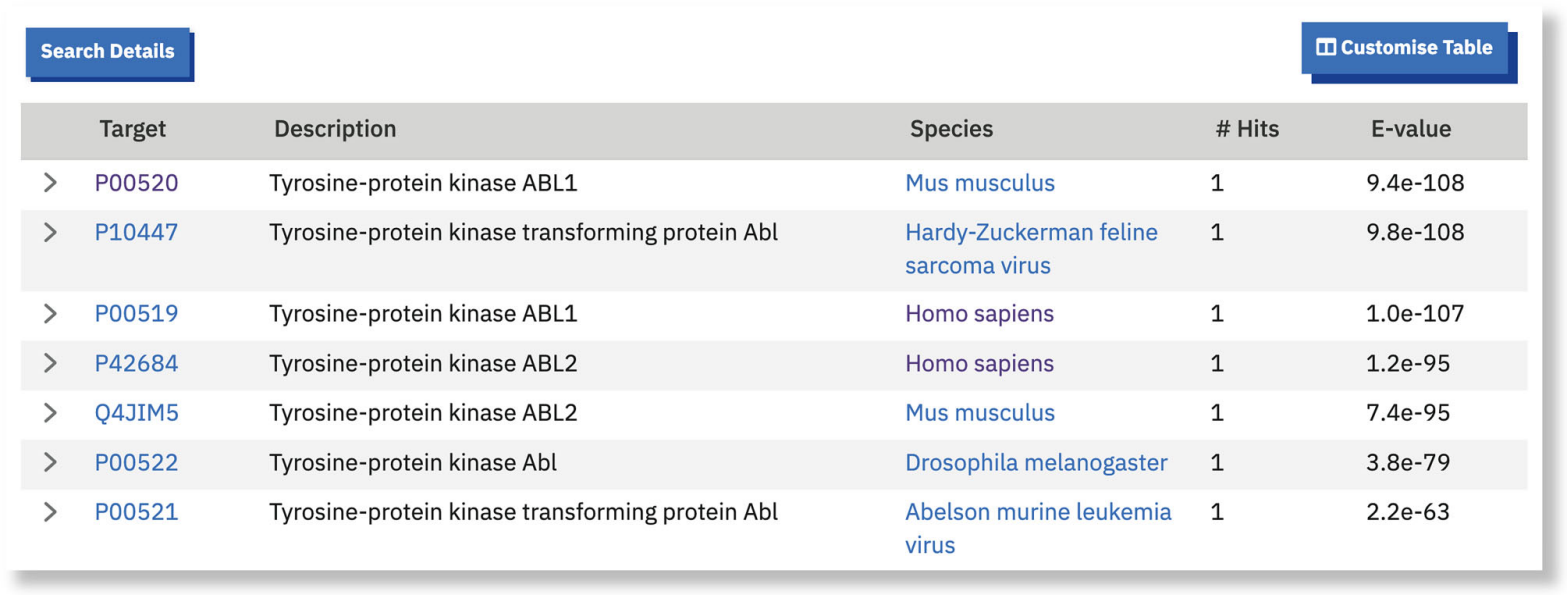
Results display with the default selection of metadata columns.

Earlier versions of the HMMER web server enriched results with extensive metadata, including links to publications in Europe PMC [25] and resources such as Gene Ontology (GO) terms [26, 27]. However, these metadata were sufficiently large that they needed to be stored in a separate metadata database (labeled “Metadata DB” in Fig. 1). This architecture introduced a performance bottleneck when merging these metadata entries with the HMMER search results. To improve performance and reliability, we removed this bottleneck by streamlining the metadata to be embedded within the *hmmpgmd*-compatible files. This allows the metadata to be returned directly with the search results. This approach could not be scaled to include the complete set of metadata previously available; these are now focused on the sequence identifiers, descriptions, and taxonomic information provided directly by the source databases (Fig. 3). Users requiring more detailed annotation can follow links to the authoritative sources (UniProt, PDB, Pfam) where comprehensive and up-to-date information is maintained. This approach reduces the complexity, while ensuring that users have access to the most current annotations available.

### Future directions

Having achieved the key goals of scalability and stability for the new HMMER server implementation, we will be re-introducing features. For example, Ensembl protein sequences will be added as a target sequence in the near future, providing access to the growing collection of species contained within Ensembl, burgeoning from global biodiversity sequencing projects. To complement the sequence annotations provided by Pfam, we plan to restore the motif predictions of coiled-coil regions (ncoils) [28], signal peptides and transmembrane domains (Phobius) [29], and intrinsically disordered regions (IUPred) [30], as well as key additional protein family databases, namely PANTHER [31] and CATH-Gene3D [18].

## Discussion

The principal achievement of this update has been the successful scaling of the HMMER web server to accommodate the continued exponential growth of protein sequence databases. This has been accomplished not through algorithmic advances—HMMER3 has remained fundamentally unchanged since 2018—but through comprehensive re-engineering of the service infrastructure. The migration to Kubernetes, the development of *hmmpgmd_shard* for database partitioning, and the introduction of an asynchronous job queue have collectively enabled the service to remain more performant and resilient to failures as databases continue to grow in scale.

The architectural approach adopted here represents a hybrid model that retains the performance advantages of in-memory database search while enabling horizontal scaling. By partitioning databases across multiple worker daemons and coordinating searches via a master process, we achieve near-linear scaling with the number of workers. The asynchronous job queue decouples the web server from the search infrastructure, improving resilience and simplifying operational tasks such as deployments and upgrades. The trade-offs, which include increased coordination complexity and a modest latency overhead, have been necessary to enable the scaling of searches. Importantly, this architecture allows further scaling as databases grow.

This infrastructure is designed to be portable and reusable beyond the public HMMER web server. For example, the Kubernetes-based deployment is planned for integration within the MGnify metagenomics analysis platform [12]. The portability of the architecture has been demonstrated through successful deployment to cloud infrastructure based on OpenStack, showing that the system can operate effectively across different orchestration environments. To facilitate community adoption, we provide a companion Nextflow pipeline that automates the database reformatting and deployment required for the HMMER web service. The modular architecture, combined with the open-source codebase, enables other groups to deploy local instances tailored to their specific needs, whether for internal use, integration with existing workflows, or providing access to custom sequence databases.

## Data Availability

The HMMER web server is available at https://www.ebi.ac.uk/Tools/hmmer. It is free and open to all users and there is no login requirement. The HMMER software suite is available at http://hmmer.org/.
